# Real-world safety profile of sonidegib: a disproportionality analysis based on the FDA adverse event reporting system

**DOI:** 10.3389/fonc.2025.1642867

**Published:** 2025-10-23

**Authors:** Kaidi Zhao, Qinxiao Li, Miao Qin, Shengxiang Xiao, Jing Li, Jiashu Liu

**Affiliations:** ^1^ Department of Dermatology, Xi’an Jiaotong University Second Affiliated Hospital, Xi’an, China; ^2^ Department of Dermatology, Xi’an Children’s Hospital, Xi’an, China

**Keywords:** adverse events, basal cell carcinoma, FAERS, sonidegib, drug safety

## Abstract

**Background:**

Sonidegib is a novel treatment for locally advanced basal cell carcinoma (LaBCC) with demonstrated efficacy and safety in clinical trials. However, its real-world safety profile remains insufficiently characterized. This study aimed to evaluate the real-world safety of sonidegib by analyzing adverse event (AE) reports from the FDA Adverse Event Reporting System (FAERS), identifying both known and unexpected safety signals.

**Methods:**

Data for this study were obtained from the FAERS, covering the period from the third quarter of 2015 to the fourth quarter of 2024. Four disproportionality analysis methods were employed to detect positive signals associated with sonidegib, including Reporting Odds Ratio (ROR), Proportional Reporting Ratio (PRR), Bayesian Confidence Propagation Neural Network (BCPNN), and Multi-item Gamma Poisson Shrinker (MGPS). Additionally, time-to-onset (TTO) analysis of AEs was conducted, and sensitivity analyses were performed to assess the robustness of the results.

**Results:**

A total of 1,087 Individual Case Safety Reports (ICSRs) involving 2,496 adverse events were included. The analysis confirmed the occurrence of known AEs, such as muscle spasms and myalgia, while also identifying several unexpected AEs, including pneumonia, sepsis, urinary tract infection, and hyperkalemia.

**Conclusions:**

This study analyzed the real-world safety of sonidegib and emphasized the importance of continuous monitoring during the early stages of treatment. These findings provide important safety information for clinicians, but further research is needed to validate these results.

## 1 Introduction

Basal cell carcinoma (BCC) is one of the most common malignancies in humans (1), exhibiting a rising global incidence (2). BCC predominantly occurs in sun-exposed areas, with the face and neck being the most frequent sites of manifestation (3). Typically, BCC presents as slowly enlarging, non-healing nodules or ulcers, which may occasionally bleed (4). Although most cases of BCC have a favorable prognosis with standard treatment regimens, the management of locally advanced basal cell carcinoma (LaBCC) remains challenging (5). The pathogenesis of BCC involves a complex interplay of genetic and environmental factors (6). Recent studies have highlighted that aberrant activation of the Hedgehog (Hh) signaling pathway plays a pivotal role in the pathogenesis of BCC (7). In BCC, the dysregulation of the Hh pathway is often linked to mutations in key oncogenic genes such as Patched 1(PTCH1) and Smoothened(SMO). These genetic alterations result in the inability to properly shut down the Hh pathway, thereby promoting tumor cell proliferation and survival (8).

Sonidegib is a novel Hh pathway inhibitor (HPI) that effectively blocks the Hh signaling pathway by targeting and inhibiting the activity of the SMO protein (9). It offers an important treatment option for patients with BCC that recurs after surgery or radiotherapy (10). However, despite demonstrating significant efficacy and a favorable safety profile in clinical trials, the spectrum of adverse events(AEs) and associated risks of sonidegib in real-world settings remain inadequately understood.

The FDA Adverse Event Reporting System (FAERS) is a voluntary reporting system designed to collect and monitor AEs associated with marketed drugs and therapeutic products (11). This database collects AE reports submitted by clinicians, pharmacists, nurses, consumers, and pharmaceutical companies, making it a valuable resource for evaluating drug safety (12). This study aims to systematically evaluate AE reports related to sonidegib using data from the FAERS. By applying multiple disproportionality analysis methods, we sought to identify both known and unexpected safety signals, thereby providing evidence-based insights to support clinical decision-making and optimize patient safety during sonidegib therapy.

## 2 Materials and methods

### 2.1 Data source and study design

**Figure 1:**
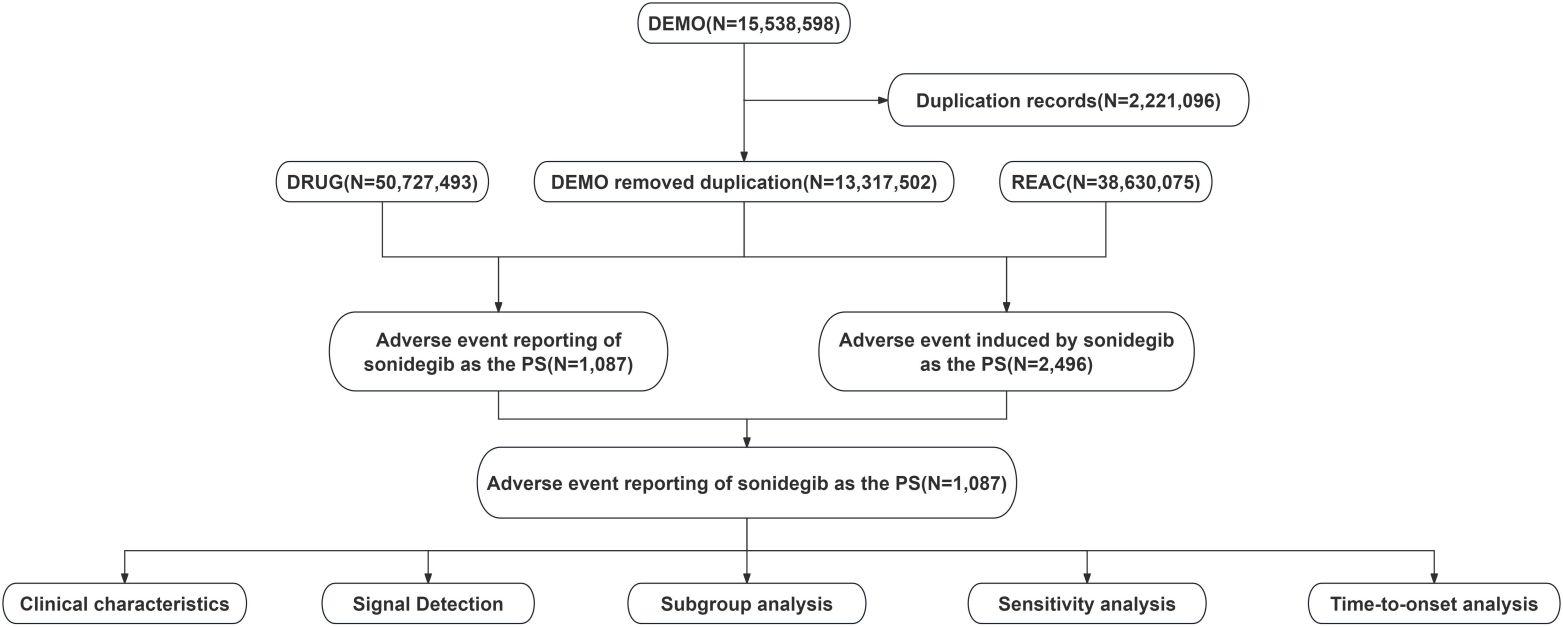
Data processing and analysis flowchart for adverse event reports associated with sonidegib from the FDA Adverse Event Reporting System (FAERS).

Data for this study were obtained from the FAERS database. In FAERS, the role of a drug in an AE report is categorized as primary suspect (PS), secondary suspect (SS), concomitant (C), or interacting (I). We collected reports from the third quarter of 2015 to the fourth quarter of 2024 in which sonidegib was identified as the PS drug. Data processing consisted mainly of two steps: deduplication and standardization of AE terminology. For deduplication, we followed FDA-recommended procedures using three parameters: Case Identifier (CASEID), FDA Receipt Date (FDA_DT), and Primary Identifier (PRIMARYID). Duplicate cases were handled as follows: (1) if the CASEID was identical, the most recent FDA_DT record was retained; (2) if both CASEID and FDA_DT were identical, the record with the highest PRIMARYID was selected. For AE terminology standardization, the Medical Dictionary for Regulatory Activities (MedDRA) version 27.1 was used, and each AE was mapped to both the System Organ Class (SOC) and Preferred Term (PT) levels to ensure consistency in classification and reporting. The methodology and data flow of the study are encapsulated in **Figure 1**, which outlines the comprehensive process from data collection to analysis.

### 2.2 Sensitivity analysis

For sensitivity analysis, we excluded reports involving three topical agents: 5-fluorouracil (5-FU), imiquimod, and mupirocin. Although they are not standard systemic therapies for laBCC, they are relatively common in real-world use. 5-FU and imiquimod are only approved for superficial BCC but are sometimes applied in laBCC; mupirocin is an antibiotic ointment mainly used for secondary infections. Because of their relatively frequent use, including these drugs could introduce heterogeneity and affect the robustness of signal detection, so they were excluded. Furthermore, to minimize the impact of systemic co-treatments, we also excluded the most commonly used antibiotics for laBCC-related infections (e.g., cephalexin, clindamycin, and doxycycline), and the corresponding reports were removed from the analysis.

### 2.3 Time-to-onset analysis of sonidegib-associated AEs

The TTO of AEs associated with sonidegib was defined as the interval between the drug initiation date recorded in the THER file and the AE occurrence date noted in the DEMO file. A detailed assessment of TTO was conducted using statistical methods, including analysis of the median and quartiles. In addition, a Weibull distribution analysis was performed to evaluate the pattern of AE occurrences.

### 2.4 Dose-stratified analysis of AEs

We explored potential dose-related patterns by examining the “dose value based on manufacturer” (DOSE_VBM) parameter in the FAERS DRUG dataset. Most DOSE_VBM entries were recorded as “unknown” (UNK). Among the identifiable doses, 440 reports were for 200 mg and 24 reports were for 400 mg, which was insufficient for disproportionality analysis. Therefore, we performed descriptive analyses of the top 20 most frequently reported AEs at the 200 mg and 400 mg doses.

### 2.5 Temporal trend analysis of AE reporting

To explore temporal trends, the observation period (2015–2024) was divided into two phases: the first 5 years (2015–2019) and the later 5 years (2020–2024). Based on the MedDRA classification system, AEs were analyzed at both the SOC and PT levels, and frequency distributions between the two periods were compared.

### 2.6 Statistical analysis

Four disproportionality analysis methods were employed in this study to identify positive safety signals associated with sonidegib. These included Reporting Odds Ratio (ROR), Proportional Reporting Ratio (PRR), Multi-Item Gamma Poisson Shrinker (MGPS), and Bayesian Confidence Propagation Neural Network (BCPNN), which were used to determine which AEs constituted positive signals. A positive signal for an AE was defined as meeting the threshold criteria of any one of the disproportionality analysis methods. The calculation methods and signal detection thresholds are detailed in **Supplementary Tables 1**, **2**. All data analyses were performed using R software (version 4.3.2).

## 3 Results

### 3.1 General characteristics

This study analyzed 1,087 reports identifying sonidegib as the primary suspect, encompassing a total of 2,496 AEs. Males accounted for 55.0% of these reports, and 38.1% involved individuals aged over 65 years. A total of 48.9% of the reports were submitted by healthcare professionals, with the majority originating from the United States (67.9%), followed by Germany (9.8%) and Italy (8.3%). Further details on the distribution of these AE reports are available in **Table 1**.

**Table 1:** Clinical characteristics of sonidegib adverse event reports from the FAERS database (Q3 2015 - Q4 2024).

Characteristics	Numbers	Case proportion (%)
Number of reports	1087	
Gender		
Male	598	55.0%
Female	367	33.8%
Missing	122	11.2%
Age		
Median (IQR)	73(62,83)	
18-64	166	15.3%
>65	414	38.1%
Missing	502	46.2%
Top 3 reported Countries		
United States	738	67.9%
Germany	106	9.8%
Italy	90	8.3%
Reporter		
Non-Healthcare professionals	458	42.1%
Healthcare professionals	531	48.9%
Missing	98	9.0%
Reporting year		
2015	8	0.7%
2016	23	2.1%
2017	26	2.4%
2018	70	6.4%
2019	79	7.3%
2020	69	6.3%
2021	72	6.6%
2022	111	10.2%
2023	127	11.7%
2024	502	46.2%

IQR, interquartile range.

### 3.2 Signal detection at the system organ classes level

**Figure 2:**
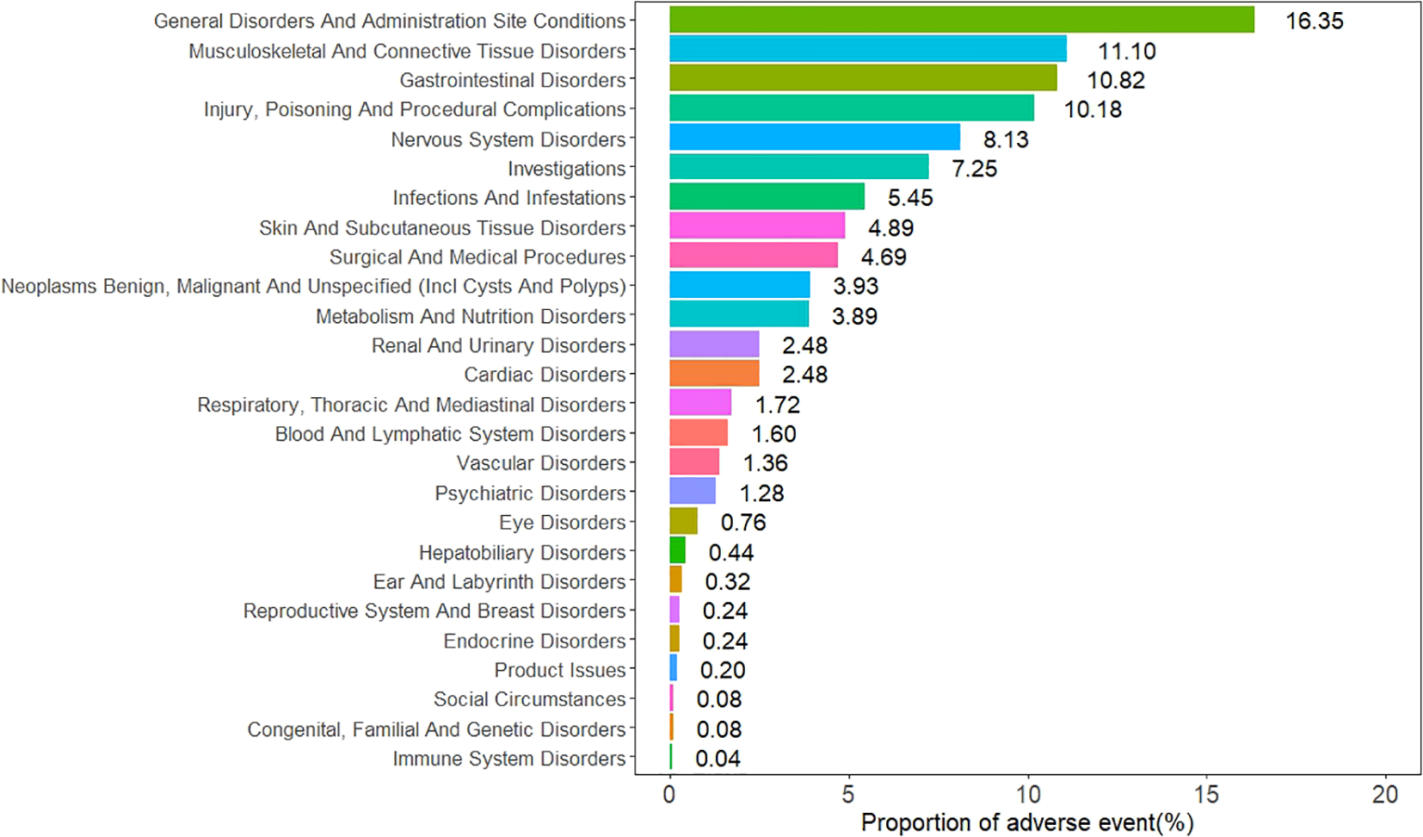
Distribution of adverse event reports by system organ class (SOC) for sonidegib.

The AEs associated with sonidegib were distributed across 26 SOCs, as illustrated in **Figure 2**. Among them, six SOCs exhibited positive safety signals, including musculoskeletal and connective tissue disorders, gastrointestinal disorders, investigations, surgical and medical procedures, neoplasms benign, malignant and unspecified (including cysts and polyps), and metabolism and nutrition disorders. The specific signal strength values for all SOCs are shown in **Table 2**.

**Table 2:** Signal strength of sonidegib-associated adverse events across system organ classes (SOC) in the FAERS database.

SOC	Numbers	ROR(95%CI)	PRR(χ^2^)	EBGM(EBGM05)	IC(IC025)
General disorders and administration site conditions	408	0.9 ( 0.81 - 1 )	0.91 ( 4.19 )	0.91 ( 0.83 )	-0.13 ( -0.29 )
Musculoskeletal and connective tissue disorders*	277	2.27 ( 2.01 - 2.58 )	2.13 ( 175.88 )	2.13 ( 1.92 )	1.09 ( 0.91 )
Gastrointestinal disorders*	270	1.34 ( 1.18 - 1.52 )	1.3 ( 20.39 )	1.3 ( 1.17 )	0.38 ( 0.19 )
Injury, poisoning and procedural complications	254	0.89 ( 0.78 - 1.01 )	0.9 ( 3.03 )	0.9 ( 0.81 )	-0.15 ( -0.34 )
Nervous system disorders	203	1.04 ( 0.9 - 1.2 )	1.03 ( 0.26 )	1.03 ( 0.92 )	0.05 ( -0.16 )
Investigations*	181	1.26 ( 1.08 - 1.47 )	1.24 ( 9.09 )	1.24 ( 1.09 )	0.31 ( 0.09 )
Infections and infestations	136	1 ( 0.84 - 1.19 )	1 ( 0 )	1 ( 0.86 )	0 ( -0.26 )
Skin and subcutaneous tissue disorders	122	0.85 ( 0.71 - 1.02 )	0.86 ( 3.16 )	0.86 ( 0.73 )	-0.23 ( -0.49 )
Surgical and medical procedures*	117	3.35 ( 2.78 - 4.04 )	3.24 ( 183.95 )	3.24 ( 2.77 )	1.7 ( 1.42 )
Neoplasms benign, malignant and unspecified (incl cysts and polyps)*	98	1.33 ( 1.09 - 1.63 )	1.32 ( 7.7 )	1.32 ( 1.11 )	0.4 ( 0.1 )
Metabolism and nutrition disorders*	97	1.94 ( 1.59 - 2.38 )	1.91 ( 42.58 )	1.91 ( 1.61 )	0.93 ( 0.63 )
Cardiac disorders	62	1.21 ( 0.94 - 1.56 )	1.21 ( 2.27 )	1.21 ( 0.98 )	0.27 ( -0.1 )
Renal and urinary disorders	62	1.29 ( 1 - 1.66 )	1.28 ( 3.91 )	1.28 ( 1.04 )	0.36 ( -0.01 )
Respiratory, thoracic and mediastinal disorders	43	0.36 ( 0.27 - 0.49 )	0.37 ( 47.95 )	0.37 ( 0.29 )	-1.43 ( -1.87 )
Blood and lymphatic system disorders	40	0.97 ( 0.71 - 1.32 )	0.97 ( 0.05 )	0.97 ( 0.74 )	-0.05 ( -0.5 )
Vascular disorders	34	0.7 ( 0.5 - 0.98 )	0.7 ( 4.34 )	0.7 ( 0.53 )	-0.51 ( -1 )
Psychiatric disorders	32	0.23 ( 0.16 - 0.33 )	0.24 ( 81.52 )	0.24 ( 0.18 )	-2.06 ( -2.57 )
Eye disorders	19	0.38 ( 0.24 - 0.59 )	0.38 ( 19.38 )	0.38 ( 0.26 )	-1.39 ( -2.04 )
Hepatobiliary disorders	11	0.53 ( 0.29 - 0.96 )	0.53 ( 4.55 )	0.53 ( 0.32 )	-0.91 ( -1.74 )
Ear and labyrinth disorders	8	0.73 ( 0.37 - 1.46 )	0.73 ( 0.78 )	0.73 ( 0.41 )	-0.45 ( -1.41 )
Reproductive system and breast disorders	6	0.33 ( 0.15 - 0.74 )	0.33 ( 7.99 )	0.33 ( 0.17 )	-1.58 ( -2.67 )
Endocrine disorders	6	0.92 ( 0.41 - 2.05 )	0.92 ( 0.04 )	0.92 ( 0.47 )	-0.12 ( -1.21 )
Product issues	5	0.11 ( 0.05 - 0.26 )	0.11 ( 36.59 )	0.11 ( 0.05 )	-3.18 ( -4.36 )
Congenital, familial and genetic disorders	2	0.29 ( 0.07 - 1.18 )	0.3 ( 3.37 )	0.3 ( 0.09 )	-1.76 ( -3.43 )
Social circumstances	2	0.18 ( 0.05 - 0.72 )	0.18 ( 7.42 )	0.18 ( 0.06 )	-2.46 ( -4.13 )
Immune system disorders	1	0.03 ( 0 - 0.23 )	0.03 ( 28.6 )	0.03 ( 0.01 )	-4.92 ( -6.96 )

Asterisks (*) indicate statistically significant signals in algorithm; ROR, reporting odds ratio; PRR, proportional reporting ratio; EBGM, empirical Bayesian geometric mean; EBGM05, the lower limit of the 95% CI of EBGM; IC, information component; IC025, the lower limit of the 95% CI of the IC; CI, confidence interval; AEs, adverse events.

### 3.3 Signal detection at the preferred terms level

**Table 3** presents the top 40 positive AEs ranked by reporting frequency. The most common AEs include muscle spasms, alopecia, fatigue, nausea, dysgeusia, diarrhea, and decreased appetite. This study confirmed several known AEs, such as muscle spasms, myalgia, alopecia, fatigue, nausea, vomiting, and decreased appetite. In addition, several unexpected AEs were identified, including pneumonia, sepsis, urinary tract infection, renal failure, arthralgia, muscular weakness, dehydration, dyspepsia, hyperkalemia, atrial fibrillation(AF) and fall.

**Table 3:** Top 40 Positive adverse events associated with sonidegib at the preferred term (PT) level.

PT	Numbers	ROR(95%CI)	PRR(c^2^)	EBGM(EBGM05)	IC(IC025)
Muscle spasms	131	18.72 ( 15.7 - 22.32 )	17.79 ( 2079.75 )	17.77 ( 15.34 )	4.15 ( 3.89 )
Off label use	83	1.91 ( 1.53 - 2.38 )	1.88 ( 34.79 )	1.88 ( 1.57 )	0.91 ( 0.59 )
Alopecia	73	8 ( 6.34 - 10.1 )	7.8 ( 433.98 )	7.79 ( 6.41 )	2.96 ( 2.62 )
Fatigue	72	2.17 ( 1.72 - 2.75 )	2.14 ( 44.27 )	2.14 ( 1.76 )	1.1 ( 0.75 )
Death	63	1.76 ( 1.37 - 2.26 )	1.74 ( 20.17 )	1.74 ( 1.41 )	0.8 ( 0.43 )
Therapy cessation	50	18.49 ( 13.97 - 24.46 )	18.14 ( 809.52 )	18.12 ( 14.33 )	4.18 ( 3.77 )
Nausea	47	1.52 ( 1.14 - 2.03 )	1.51 ( 8.26 )	1.51 ( 1.19 )	0.6 ( 0.18 )
Asthenia	43	2.93 ( 2.17 - 3.96 )	2.9 ( 53.8 )	2.9 ( 2.25 )	1.54 ( 1.1 )
Ageusia	43	46.3 ( 34.23 - 62.62 )	45.52 ( 1867.57 )	45.39 ( 35.26 )	5.5 ( 5.06 )
Product dose omission issue	41	3.08 ( 2.26 - 4.2 )	3.05 ( 56.67 )	3.05 ( 2.35 )	1.61 ( 1.16 )
Myalgia	40	6.28 ( 4.6 - 8.59 )	6.2 ( 174.78 )	6.2 ( 4.77 )	2.63 ( 2.18 )
Decreased appetite	39	4.03 ( 2.93 - 5.52 )	3.98 ( 87.3 )	3.98 ( 3.05 )	1.99 ( 1.53 )
Blood creatine phosphokinase increased	39	47.09 ( 34.3 - 64.64 )	46.37 ( 1726.56 )	46.23 ( 35.47 )	5.53 ( 5.07 )
Weight decreased	38	3.38 ( 2.45 - 4.65 )	3.34 ( 62.54 )	3.34 ( 2.55 )	1.74 ( 1.27 )
Dysgeusia	34	12.31 ( 8.78 - 17.28 )	12.16 ( 348.34 )	12.15 ( 9.15 )	3.6 ( 3.11 )
Vomiting	32	1.81 ( 1.28 - 2.57 )	1.8 ( 11.56 )	1.8 ( 1.35 )	0.85 ( 0.35 )
Therapy interrupted	29	8.88 ( 6.16 - 12.81 )	8.79 ( 200.28 )	8.78 ( 6.46 )	3.13 ( 2.6 )
Product use issue	29	2.9 ( 2.01 - 4.18 )	2.87 ( 35.55 )	2.87 ( 2.11 )	1.52 ( 0.99 )
Disease progression	27	5.65 ( 3.86 - 8.25 )	5.59 ( 102.05 )	5.59 ( 4.07 )	2.48 ( 1.94 )
Arthralgia	26	1.49 ( 1.01 - 2.19 )	1.48 ( 4.12 )	1.48 ( 1.07 )	0.57 ( 0.01 )
Fall	24	1.82 ( 1.22 - 2.73 )	1.82 ( 8.85 )	1.82 ( 1.3 )	0.86 ( 0.28 )
Muscular weakness	23	5.38 ( 3.57 - 8.11 )	5.34 ( 81.21 )	5.34 ( 3.79 )	2.42 ( 1.82 )
Pneumonia	22	1.68 ( 1.1 - 2.55 )	1.67 ( 5.96 )	1.67 ( 1.18 )	0.74 ( 0.14 )
Malignant neoplasm progression	21	4.55 ( 2.96 - 7 )	4.52 ( 57.72 )	4.52 ( 3.16 )	2.18 ( 1.56 )
Taste disorder	20	19.71 ( 12.69 - 30.61 )	19.56 ( 351.94 )	19.54 ( 13.52 )	4.29 ( 3.66 )
Urinary tract infection	19	2.71 ( 1.72 - 4.25 )	2.69 ( 20.31 )	2.69 ( 1.85 )	1.43 ( 0.78 )
Basal cell carcinoma	18	27.35 ( 17.19 - 43.49 )	27.16 ( 452.8 )	27.11 ( 18.39 )	4.76 ( 4.1 )
Febrile neutropenia	17	6.25 ( 3.88 - 10.07 )	6.22 ( 74.46 )	6.21 ( 4.17 )	2.64 ( 1.95 )
Inappropriate schedule of product administration	16	1.76 ( 1.08 - 2.88 )	1.75 ( 5.21 )	1.75 ( 1.16 )	0.81 ( 0.11 )
Therapeutic product effect incomplete	15	3.8 ( 2.29 - 6.31 )	3.78 ( 30.73 )	3.78 ( 2.47 )	1.92 ( 1.19 )
Dehydration	14	2.97 ( 1.75 - 5.02 )	2.95 ( 18.14 )	2.95 ( 1.9 )	1.56 ( 0.82 )
Sepsis	13	3.03 ( 1.75 - 5.22 )	3.02 ( 17.54 )	3.01 ( 1.91 )	1.59 ( 0.82 )
Dyspepsia	11	3 ( 1.66 - 5.42 )	2.99 ( 14.59 )	2.99 ( 1.82 )	1.58 ( 0.75 )
Hemorrhage	11	2.68 ( 1.48 - 4.85 )	2.68 ( 11.55 )	2.67 ( 1.63 )	1.42 ( 0.58 )
Renal failure	10	1.91 ( 1.03 - 3.56 )	1.91 ( 4.32 )	1.91 ( 1.13 )	0.93 ( 0.06 )
Blood creatinine increased	10	4.28 ( 2.3 - 7.97 )	4.27 ( 25.05 )	4.27 ( 2.54 )	2.09 ( 1.22 )
Hyperkalemia	9	7.04 ( 3.66 - 13.55 )	7.02 ( 46.45 )	7.02 ( 4.06 )	2.81 ( 1.9 )
Atrial fibrillation	8	2.13 ( 1.06 - 4.26 )	2.13 ( 4.78 )	2.13 ( 1.19 )	1.09 ( 0.12 )
Hematuria	8	6.03 ( 3.01 - 12.08 )	6.02 ( 33.48 )	6.02 ( 3.37 )	2.59 ( 1.63 )
Triple negative breast cancer	8	366.4 ( 181.54 - 739.47 )	365.22 ( 2838.66 )	356.8 ( 198.27 )	8.48 ( 7.5 )

ROR, reporting odds ratio; PRR, proportional reporting ratio; EBGM, empirical Bayesian geometric mean; EBGM05, the lower limit of the 95% CI of EBGM; IC, information component; IC025, the lower limit of the 95% CI of the IC; CI, confidence interval; PT, preferred term.

### 3.4 Subgroup analysis

Among male patients, the most commonly reported AEs included muscle spasms, fatigue, alopecia, arthralgia, and muscular weakness. In female patients, the most frequent AEs were muscle spasms, alopecia, fatigue, fall, and urinary tract infection. Detailed information is provided in **Supplementary Tables 3**, **4**. In patients aged 18 to 64 years, the most common AEs included muscle spasms, alopecia, fatigue, and dyspepsia. For patients over 65 years of age, the most frequently reported AEs were muscle spasms, blood creatine phosphokinase increased, fatigue, urinary tract infection, fall, and sepsis. The detailed data can be found in **Supplementary Tables 5**, **6**.

### 3.5 Sensitivity analysis

Sonidegib is typically administered as monotherapy for the treatment of LaBCC in clinical practice. However, it may also be co-administered with certain topical or systemic agents. After excluding reports involving the most commonly co-administered drugs with sonidegib, a total of 1,078 AE reports were included in the analysis, and disproportionality was re-evaluated. AEs that continued to exhibit positive signals included muscle spasms, pneumonia, sepsis, urinary tract infection, renal failure, arthralgia, muscular weakness, dehydration, dyspepsia, hyperkalemia, atrial fibrillation, and fall. Signal strength values for the top 40 positive AEs are presented in **Supplementary Table 7**.

### 3.6 Time-to-onset of sonidegib associated AEs

**Figure 3:**
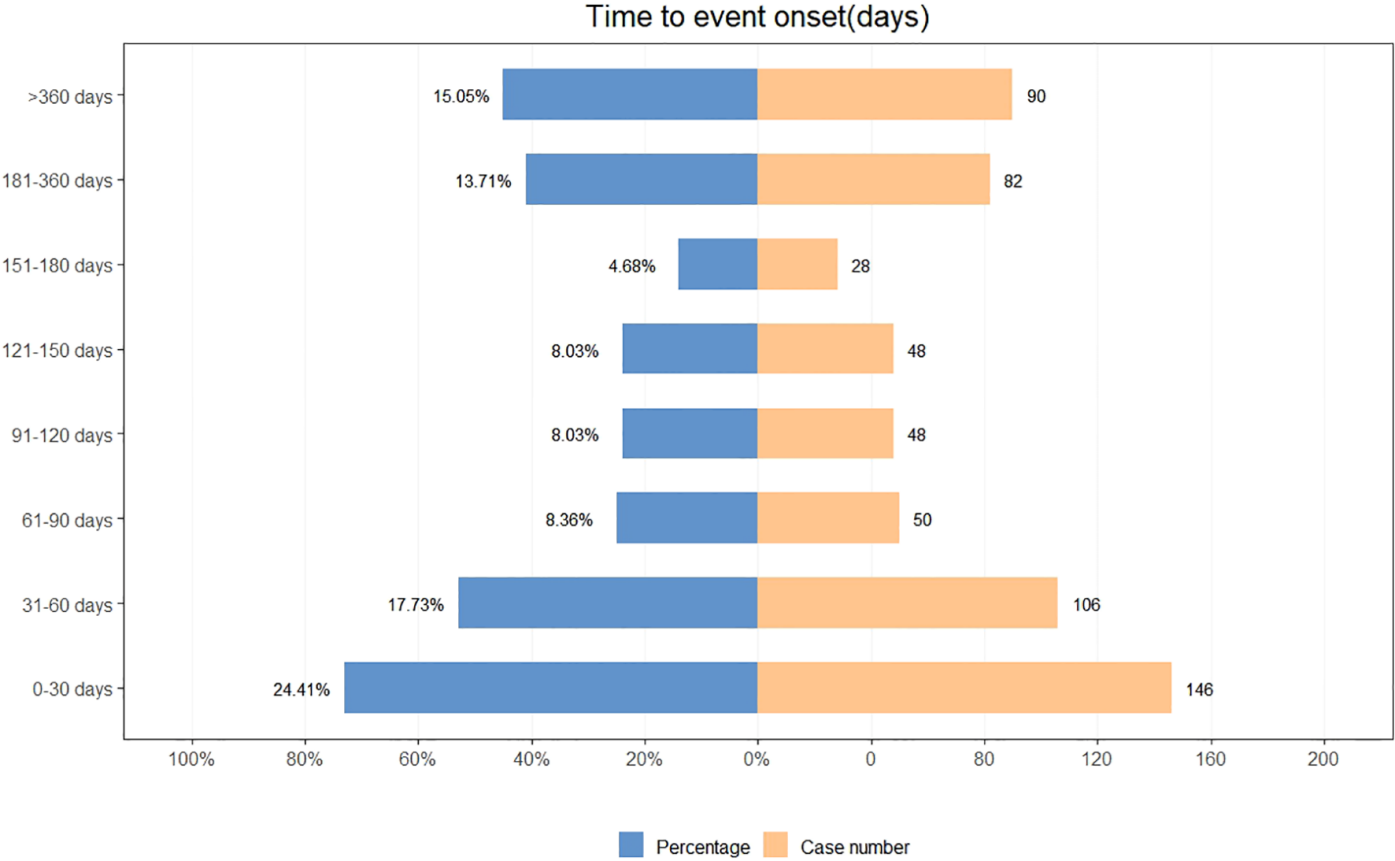
Time to event onset for adverse events associated with sonidegib.

**Figure 4:**
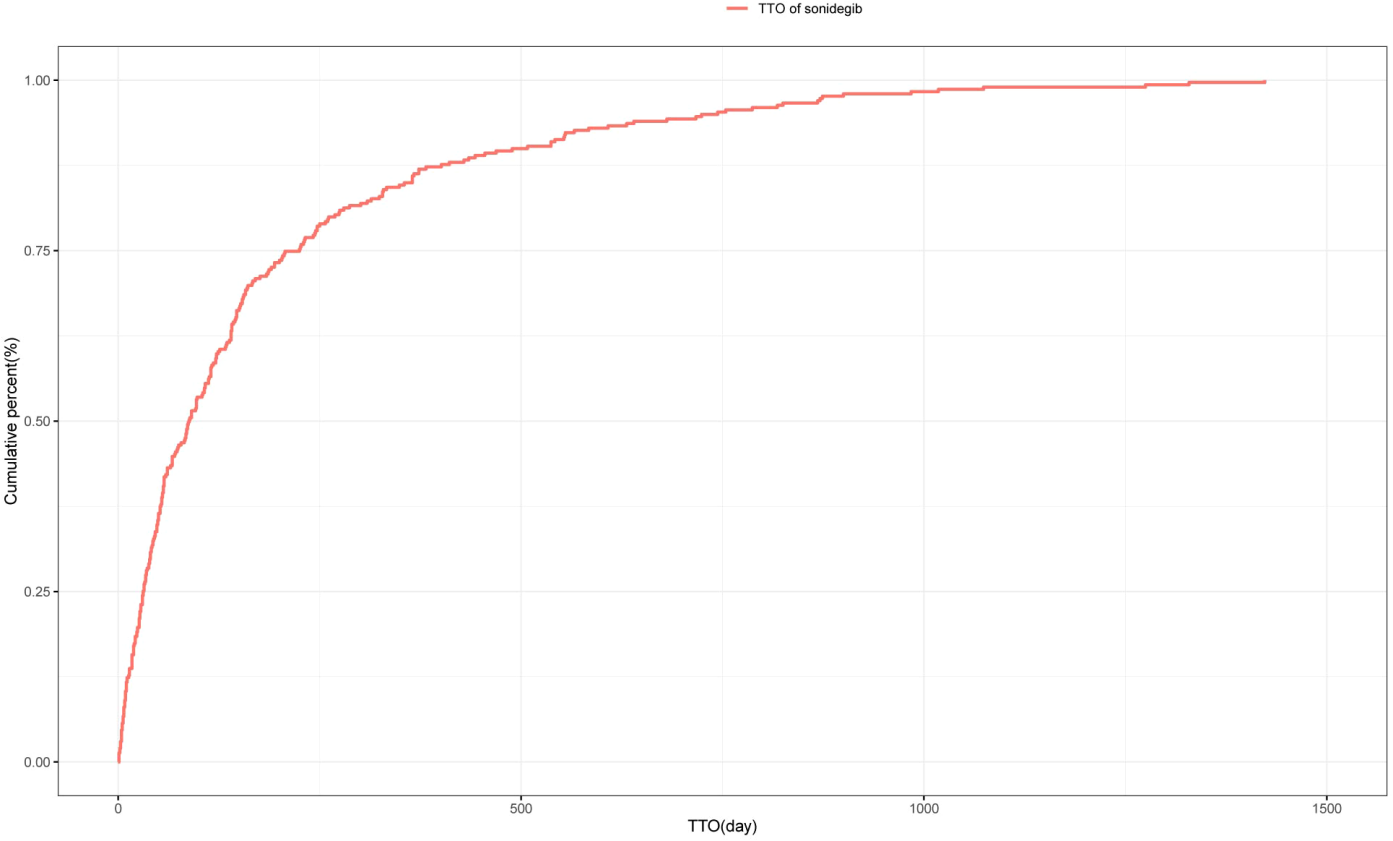
Cumulative incidence of adverse events over time for sonidegib.

A total of 598 reports provided detailed information on the TTO of AEs, with their distribution shown in **Figure 3**. Approximately 24.4% of AEs occurred within the first month after treatment initiation, and the median TTO was 88 days. The cumulative distribution of these onset times is illustrated in **Figure 4**. In addition, a Weibull distribution analysis was performed on the TTO data for sonidegib-related AEs. The results indicated an early failure model, as presented in **Table 4**.

**Table 4:** Time to onset of sonidegib-associated adverse events and results of Weibull distribution analysis.

Drug	TTO(days)		Weibull distribution		
	Case reports	Median(d)(IQR)	Scale parameter: α(95%CI)	Shape parameter: β(95%CI)	Type
Sonidegib	598	88(31.25,220.50)	153.36(136.73,169.98)	0.78(0.73,0.83)	Early failure

TTO, time to onset; CI, confidence interval; IQR, interquartile range.

### 3.7 AE profiles by sonidegib dose

At the FDA-approved 200 mg dose group (n = 440), the most frequently reported AEs included muscle spasms, fatigue, alopecia, increased blood creatine phosphokinase, decreased appetite, dysgeusia, and muscular weakness, which are consistent with the typical Hedgehog pathway inhibitor–related toxicities observed in clinical trials. In the 400 mg dose group (n = 24), AEs were more often associated with severe clinical outcomes, such as sepsis, febrile neutropenia, and cardiac failure, whereas no clear clustering of muscle spasms or dysgeusia was observed. The top 20 most frequently reported AEs for these two doses are presented in **Supplementary Tables 8**, **9**.

### 3.8 Temporal patterns of AEs across study periods

To explore temporal trends, we divided the observation period into the first 5 years (2015–2019) and the later 5 years (2020–2024). At the SOC level, both periods were dominated by General disorders and administration site conditions, Gastrointestinal disorders, Musculoskeletal and connective tissue disorders, and Nervous system disorders, suggesting overall stability in distribution. However, Immune system disorders appeared only in the first 5 years (n=1), while Endocrine disorders, Congenital, familial and genetic disorders, and Social circumstances emerged in the later period (**Supplementary Figures 1**, **2**). At the PT level, high-frequency AEs in the earlier period were more concentrated on serious outcomes such as malignant neoplasm progression, febrile neutropenia, and sepsis. By contrast, the later period showed higher frequencies of drug-related toxicities and adherence issues such as muscle spasms, dysgeusia, blood creatine phosphokinase increased, and therapy cessation. Notably, muscle spasms, alopecia, and fatigue remained frequent in both periods, suggesting they represent persistent core safety signals of sonidegib (**Supplementary Tables 10**, **11**).

## 4 Discussion

Based on data from the FAERS database, this study systematically evaluated AEs reported since the approval of sonidegib. In addition to the AEs documented in the drug label, sonidegib may pose further safety concerns, including sepsis, urinary tract infection, renal failure, hyperkalemia, AF and fall. Notably, 24.4% of AEs occurred within the first month of treatment initiation. These findings offer preliminary insight into the real-world safety profile of sonidegib and emphasize the need for early identification and proactive management of potential risks in clinical practice.

At the SOC level, our study indicated that the most commonly involved categories were musculoskeletal and connective tissue disorders, gastrointestinal disorders, investigations, and metabolism and nutrition disorders. This aligned with the outcomes of previous clinical trials for the treatment of BCC (13). At the PT level, sonidegib caused AEs listed in the drug label, including muscle spasms, myalgia, alopecia, fatigue, nausea, vomiting and decreased appetite (14). In one clinical trial, 49% of participants experienced muscle spasms with a daily dose of 200mg of Sonidegib, which increased to 67% at a daily dose of 800mg (15). Another long-term trial lasting 42 months observed muscle spasms at rates of 54.4% and 69.3% for the 200mg and 800mg doses of sonidegib, respectively (16). Although most muscle spasms were mild or moderate, their frequent occurrence at night or during rest could disrupt treatment for many patients (17). The specific mechanism behind muscle spasms remains unclear, but it is thought to be related to the inhibition of the canonical SMO pathway and the activation of the non-canonical SMO/Ca+/AMPK pathway, leading to an influx of calcium and resultant muscle contractions (18). Given the significant impact of muscle spasms on patients’ quality of life and the potential to lead to treatment discontinuation, careful monitoring during drug use is necessary. Additionally, other AEs listed in the drug label also warranted attention as they imposed physical and psychological burdens on patients, potentially adversely affecting the therapeutic efficacy of the drug (19).

Notably, our study identified unexpected AEs associated with sonidegib, particularly concerning infections and infestations, as well as renal and urinary disorders. Further analysis at the PT level revealed positive signals for pneumonia, sepsis, and urinary tract infection. The Hedgehog signaling pathway is a transduction signaling cascade that regulates various cellular functions (20), including the proliferation, migration, differentiation, and survival of cells during embryonic development and plays a significant role in cancer progression (21). Recent studies have also shown that the Hedgehog pathway continues to function in adult tissues, participating in the differentiation, proliferation, and functional regulation of macrophages, NKT cells, T cells, and B cells (22). Multiple studies have reported that Sonic hedgehog (Shh) signaling plays a crucial role in the proliferation of CD4+ T cells (22). As a SMO inhibitor, sonidegib suppresses a key molecule in the Hedgehog pathway, which could hypothetically modulate immune function and increase susceptibility to infections in some patients. However, this mechanistic explanation is speculative and not directly supported by the present dataset. In addition, infections such as pneumonia, sepsis, and urinary tract infection are also strongly influenced by baseline patient factors including frailty, comorbidities, and advanced age, which may predispose to these events independently of drug exposure. Because FAERS does not consistently capture these variables, residual confounding cannot be excluded. The observed signals should therefore be interpreted as statistical associations rather than evidence of causation. The role of the Hedgehog signaling pathway in immune responses is not yet fully understood and requires further exploration.

In addition, sonidegib has been associated with previously unreported AEs, such as renal failure and hyperkalemia. The Hh signaling pathway has been shown to play a protective role in maintaining renal function (23). By inhibiting the Hh pathway, sonidegib may interfere with these protective effects, thereby potentially leading to renal failure. It is important to emphasize that our study only demonstrated a statistical association between sonidegib and renal failure, rather than a causal relationship, and prospective studies are needed to validate these findings. Nevertheless, such injuries pose a serious threat to patient survival, and healthcare professionals should remain highly vigilant.

Disproportionality analysis suggested a statistical association between sonidegib and falls, possibly related to lower limb muscle weakness. However, this event has never previously been reported as directly related to sonidegib in clinical studies, and the finding should therefore be interpreted as a safety signal rather than proof of causation. AF typically presents with palpitations, dizziness, and chest tightness, along with irregular heart rhythm, a heart rate higher than the pulse rate, and variable intensity of heart sounds (24, 25). AF may lead to serious complications such as depression, heart failure, cardiomyopathy, and ischemic stroke (26). In our analysis, AF emerged as a positive signal, suggesting a potential statistical association with sonidegib. However, to date, AF has not been directly reported in clinical studies as related to sonidegib. The association between sonidegib and AF therefore remains speculative, and our findings do not provide direct evidence of a causal relationship. This signal should thus be interpreted with caution. Future prospective studies are warranted to further validate this observation.

After excluding reports involving the concomitant use of sonidegib with 5-fluorouracil, imiquimod, or mupirocin, a repeated disproportionality analysis was performed. The results indicated that AEs such as muscle spasms, pneumonia, sepsis, urinary tract infection, renal failure, arthralgia, muscular weakness, dehydration, dyspepsia, hyperkaliemia, AF, and falls continued to exhibit positive signals. These findings further reinforce the robustness and reliability of the results.

Muscle spasms, fatigue, and alopecia were common AEs across different genders. In females, particular attention should be given to the occurrence of falls and urinary tract infections. Muscle spasms and fatigue were frequently reported across all age groups. In patients aged over 65 years, urinary tract infections, falls, and sepsis should be closely monitored. The frequency of AEs across different subgroups warrants further large-scale studies for exploration.

TTO analysis showed that approximately 24.4% of AEs were reported within the first month after treatment initiation, with a median TTO of 88 days. However, this pattern differs from clinical trial data (27, 28) (such as the BOLT trial and its long-term extension studies), in which many AEs (such as muscle spasms) tended to occur after several months of treatment. Therefore, the early clustering observed in this study should be interpreted with caution, as it may reflect reporting bias, incomplete information, or heightened awareness during the initial phase of therapy, particularly given that a considerable proportion of FAERS reports were submitted by non-healthcare professionals. Taken together, our findings do not suggest that the first month carries a uniquely high risk, but rather emphasize the importance of maintaining vigilance for AEs from the start of treatment and throughout the entire therapeutic process. Future prospective studies are still needed to further clarify the temporal distribution pattern of sonidegib-related AEs and to provide stronger evidence for clinical practice.

These findings suggest that sonidegib at the FDA-approved 200 mg dose is primarily associated with typical Hedgehog pathway–related toxicities, such as muscle spasms, fatigue, alopecia, increased blood creatine phosphokinase, decreased appetite, dysgeusia, and muscular weakness, which is consistent with clinical trial results. In contrast, the 400 mg dose appeared to be more frequently linked with severe clinical outcomes, including sepsis, febrile neutropenia, and cardiac failure, while no clear clustering of muscle spasms or dysgeusia was observed. This pattern may indicate reduced tolerability at higher doses. However, given the very limited number of 400 mg reports, the observed differences should be interpreted with caution.

The temporal trend analysis provides further insights into the evolving safety profile of sonidegib. Although the overall SOC distribution remained relatively stable, changes at the PT level suggest significant shifts in AE reporting patterns. In the early years following approval, reports were more focused on serious outcomes such as neutropenia and sepsis, reflecting heightened clinical vigilance toward life-threatening events and limited prior experience. In contrast, in the later period, reports increasingly captured drug-related toxicities (e.g., muscle spasms, dysgeusia, blood creatine phosphokinase increased) and adherence issues (e.g., therapy cessation), reflecting accumulated experience, improved recognition of typical toxicities, and broader patient exposure. This phenomenon may be partly explained by changes in reporting practices and learning effects: with increasing familiarity with the safety profile of sonidegib, clinicians and patients became more likely to report characteristic AEs, whereas the relative proportion of serious outcomes decreased. In addition, the emergence of new potential safety signals (e.g., dysgeusia, blood creatine phosphokinase increased) in the later period highlights risks that may not have been fully identified in clinical trials. Overall, the temporal trend analysis underscores the importance of continuous pharmacovigilance throughout the drug lifecycle, as AE reporting patterns may evolve from early emphasis on serious outcomes to later focus on long-term toxicities and adherence issues.

There are several limitations in this study. First, the FAERS database is based on spontaneous reports submitted by clinicians, pharmacists, nurses, and consumers, which inherently introduces potential reporting bias. In particular, nearly half of the reports (42.1%) were submitted by non-healthcare professionals. While such reports broaden the scope of patient-reported outcomes, they may lack the clinical accuracy and detail typically provided by trained healthcare professionals. This limitation could lead not only to misclassification and incomplete information, but also to substantial bias in the observed frequencies of AEs and in the strength of disproportionality signals. Another limitation of this study is the potential impact of concomitant medications. Although we excluded commonly used topical agents (5-FU, imiquimod, mupirocin) and several antibiotics frequently prescribed for laBCC-related infections (cephalexin, clindamycin, doxycycline), other systemic co-treatments may still exist and interfere with the attribution of AEs. In addition, the FAERS database does not consistently provide complete information on concomitant therapies, drug dosage, or timing of administration, making it impossible to completely rule out residual confounding. These limitations may affect the observed signal strength and attribution of AEs. It is noteworthy that a considerable proportion of reports lacked demographic information, with 46.2% missing age and approximately 11% missing gender. Such incompleteness may introduce reporting bias and restrict the interpretation of disproportionality analyses, particularly for subgroup analyses by age and sex. A key limitation of this study is the lack of dose information in FAERS. Most reports did not provide DOSE_VBM data, and the number of higher-dose reports (e.g., 400 mg) was too small to allow for disproportionality analyses. As a result, only descriptive analyses were feasible. Although multiple disproportionality analyses identified several unexpected AEs, these findings do not establish a causal relationship between sonidegib and these events but rather indicate a statistical association. Further prospective studies are needed to confirm these signals. Finally, the majority of AE reports originated from the United States, with relatively few reports from other regions, which may limit the external validity and generalizability of the findings across diverse populations.

## 5 Conclusions

This study conducted a disproportionality analysis of AEs associated with sonidegib using data from the FAERS database. In addition to the AEs listed on the drug label, several unexpected AEs were identified, including pneumonia, sepsis, UTI, and hyperkalemia. The study further emphasizes the importance of safety monitoring during sonidegib treatment. These findings provide real-world insights into the safety profile of sonidegib, thereby supporting its safe and rational use by clinicians. However, given the inherent limitations of FAERS, including reporting bias, incomplete clinical information, and potential confounding by concomitant medications, these results should be interpreted as statistical signals rather than evidence of causation. Further prospective studies are warranted to validate these observations.

## Data Availability

Publicly available datasets were analyzed in this study. This data can be found here: The database used in this study can be accessed at: https://fis.fda.gov/extensions/FPD-QDE-FAERS/FPD-QDE-FAERS.html.
