# Supplementary material for: Real-world safety profile of sonidegib: a disproportionality analysis based on the FDA adverse event reporting system
Source: Front Oncol. 2025 Oct 23;15:1642867. doi: 10.3389/fonc.2025.1642867 (PMC12588830; doi:10.3389/fonc.2025.1642867)
Supplement: Supplementary file 1 [file DataSheet1.docx]

Supplementary Material

Supplementary Tables

**Supplementary Table 1:**

**Two-by-two contingency table for disproportionality analyses**

|  | Target AEs | Other AEs | Total |
| --- | --- | --- | --- |
| Sonidegib | a | b | a+b |
| Other drugs | c | d | c+d |
| Total | a+c | b+d | a+b+c+d |

Abbreviation: AEs, adverse events; a, number of reports containing both the target drug and target adverse drug reaction; b, number of reports containing other adverse drug reaction of the target drug; c, number of reports containing the target adverse drug reaction of other drugs; d, number of reports containing other drugs and other adverse drug reactions.

**Supplementary Table 2:**

**Four major algorithms used for signal detection**

| Algorithms | Equation | Criteria |
| --- | --- | --- |
| ROR | ROR=ad/b/c | lower limit of 95% CI>1, a≥3 |
|  | 95%CI=e^ln(ROR)±1.96(1/a+1/b+1/c+1/d)^0.5^ |  |
| PRR | PRR=a(c+d)/c/(a+b) | PRR≥2, χ^2^≥4, a≥3 |
|  | χ^2^=[(ad-bc)^2](a+b+c+d)/[(a+b)(c+d)(a+c)(b+d)] |  |
| BCPNN | IC=log_2_a(a+b+c+d)(a+c)(a+b) | IC025>0 |
|  | 95%CI= E(IC) ± 2V(IC)^0.5 |  |
| MGPS | EBGM=a(a+b+c+d)/(a+c)/(a+b) | EBGM05>2 |
|  | 95%CI=e^ln(EBGM)±1.96(1/a+1/b+1/c+1/d)^0.5^ |  |

Abbreviation: a, number of reports containing both the target drug and target adverse drug reaction; b, number of reports containing other adverse drug reaction of the target drug; c, number of reports containing the target adverse drug reaction of other drugs; d, number of reports containing other drugs and other adverse drug reactions. 95%CI, 95% confidence interval; N, the number of reports; χ2, chi-squared; IC, information component; IC025, the lower limit of 95% CI of the IC; E(IC), the IC expectations; V(IC), the variance of IC; EBGM, empirical Bayesian geometric mean; EBGM05, the lower limit of 95% CI of EBGM.

**Supplementary Table 3:**

**Top 20 Most Frequent Adverse Events for Sonidegib in Males at the Preferred Term (PT) Level from FAERS Data**

| PT | Numbers | ROR(95%Cl) | PRR | EBGM(EBGM05) | IC(IC025) |
| --- | --- | --- | --- | --- | --- |
| Muscle spasms* | 84 | 24.84 ( 19.92 - 30.97 ) | 23.45 ( 1805.01 ) | 23.39 ( 19.45 ) | 4.55 ( 4.23 ) |
| Fatigue* | 45 | 2.53 ( 1.88 - 3.41 ) | 2.48 ( 40.43 ) | 2.48 ( 1.94 ) | 1.31 ( 0.88 ) |
| Alopecia* | 44 | 31.06 ( 23 - 41.95 ) | 30.14 ( 1236.64 ) | 30.04 ( 23.36 ) | 4.91 ( 4.47 ) |
| Death | 36 | 1.28 ( 0.92 - 1.78 ) | 1.27 ( 2.13 ) | 1.27 ( 0.96 ) | 0.35 ( -0.13 ) |
| Ageusia* | 29 | 45.43 ( 31.43 - 65.68 ) | 44.54 ( 1228.64 ) | 44.32 ( 32.56 ) | 5.47 ( 4.94 ) |
| Asthenia* | 28 | 3.08 ( 2.12 - 4.48 ) | 3.04 ( 38.67 ) | 3.04 ( 2.23 ) | 1.61 ( 1.06 ) |
| Weight decreased* | 27 | 3.63 ( 2.48 - 5.32 ) | 3.58 ( 50.54 ) | 3.58 ( 2.6 ) | 1.84 ( 1.29 ) |
| Therapy cessation* | 27 | 16.47 ( 11.25 - 24.11 ) | 16.18 ( 384.3 ) | 16.15 ( 11.74 ) | 4.01 ( 3.46 ) |
| Nausea* | 25 | 1.92 ( 1.29 - 2.85 ) | 1.91 ( 10.85 ) | 1.91 ( 1.37 ) | 0.93 ( 0.36 ) |
| Blood creatine phosphokinase increased* | 24 | 30.77 ( 20.54 - 46.09 ) | 30.27 ( 677.42 ) | 30.17 ( 21.52 ) | 4.92 ( 4.33 ) |
| Product dose omission issue* | 24 | 3.2 ( 2.13 - 4.79 ) | 3.16 ( 35.6 ) | 3.16 ( 2.25 ) | 1.66 ( 1.08 ) |
| Decreased appetite* | 22 | 3.51 ( 2.3 - 5.35 ) | 3.47 ( 38.87 ) | 3.47 ( 2.44 ) | 1.8 ( 1.19 ) |
| Diarrhoea | 22 | 1.42 ( 0.93 - 2.17 ) | 1.41 ( 2.7 ) | 1.41 ( 0.99 ) | 0.5 ( -0.11 ) |
| Myalgia* | 21 | 6.01 ( 3.91 - 9.25 ) | 5.94 ( 86.43 ) | 5.94 ( 4.14 ) | 2.57 ( 1.95 ) |
| Drug ineffective | 20 | 0.64 ( 0.41 - 0.99 ) | 0.64 ( 4 ) | 0.64 ( 0.45 ) | -0.63 ( -1.27 ) |
| Therapy interrupted* | 19 | 10.16 ( 6.46 - 15.98 ) | 10.04 ( 154.64 ) | 10.03 ( 6.86 ) | 3.33 ( 2.68 ) |
| Arthralgia* | 18 | 2.24 ( 1.41 - 3.57 ) | 2.23 ( 12.25 ) | 2.23 ( 1.51 ) | 1.16 ( 0.49 ) |
| Vomiting* | 18 | 2.19 ( 1.37 - 3.48 ) | 2.17 ( 11.44 ) | 2.17 ( 1.47 ) | 1.12 ( 0.45 ) |
| Dysgeusia* | 18 | 12.85 ( 8.07 - 20.46 ) | 12.7 ( 193.93 ) | 12.68 ( 8.59 ) | 3.66 ( 3 ) |
| Muscular weakness* | 18 | 7.29 ( 4.58 - 11.6 ) | 7.21 ( 96.36 ) | 7.2 ( 4.88 ) | 2.85 ( 2.18 ) |

Abbreviation: Asterisks (*) indicate statistically significant signals in algorithm; ROR, reporting odds ratio; PRR, proportional reporting ratio; EBGM, empirical Bayesian geometric mean; EBGM05, the lower limit of the 95% CI of EBGM; IC, information component; IC025, the lower limit of the 95% CI of the IC; CI, confidence interval; PT,preferred term; AEs, adverse events.

**Supplementary Table 4:**

**Top 20 Most Frequent Adverse Events for Sonidegib in Females at the Preferred Term (PT) Level from FAERS Data**

| PT | Numbers | ROR(95%Cl) | PRR | EBGM(EBGM05) | IC(IC025) |
| --- | --- | --- | --- | --- | --- |
| Muscle spasms* | 38 | 13.05 ( 9.43 - 18.06 ) | 12.54 ( 404.73 ) | 12.53 ( 9.55 ) | 3.65 ( 3.18 ) |
| Alopecia* | 26 | 5.77 ( 3.91 - 8.53 ) | 5.63 ( 99.59 ) | 5.63 ( 4.06 ) | 2.49 ( 1.93 ) |
| Fatigue* | 26 | 2.04 ( 1.38 - 3.01 ) | 2.01 ( 13.37 ) | 2.01 ( 1.45 ) | 1.01 ( 0.44 ) |
| Nausea* | 21 | 1.59 ( 1.03 - 2.46 ) | 1.58 ( 4.54 ) | 1.58 ( 1.1 ) | 0.66 ( 0.04 ) |
| Death* | 21 | 2.36 ( 1.53 - 3.64 ) | 2.33 ( 16.07 ) | 2.33 ( 1.62 ) | 1.22 ( 0.6 ) |
| Therapy cessation* | 18 | 17.81 ( 11.17 - 28.41 ) | 17.47 ( 279.64 ) | 17.46 ( 11.81 ) | 4.13 ( 3.46 ) |
| Decreased appetite* | 17 | 5.07 ( 3.14 - 8.19 ) | 4.99 ( 54.45 ) | 4.99 ( 3.34 ) | 2.32 ( 1.63 ) |
| Myalgia* | 16 | 6.59 ( 4.02 - 10.81 ) | 6.49 ( 74.52 ) | 6.49 ( 4.29 ) | 2.7 ( 1.99 ) |
| Asthenia* | 14 | 2.63 ( 1.55 - 4.46 ) | 2.61 ( 13.95 ) | 2.61 ( 1.68 ) | 1.38 ( 0.63 ) |
| Vomiting* | 14 | 1.95 ( 1.15 - 3.31 ) | 1.93 ( 6.37 ) | 1.93 ( 1.24 ) | 0.95 ( 0.2 ) |
| Diarrhoea | 14 | 1.35 ( 0.79 - 2.28 ) | 1.34 ( 1.23 ) | 1.34 ( 0.86 ) | 0.42 ( -0.33 ) |
| Dysgeusia* | 14 | 12.79 ( 7.54 - 21.68 ) | 12.6 ( 149.63 ) | 12.6 ( 8.1 ) | 3.65 ( 2.9 ) |
| Product dose omission issue* | 14 | 2.89 ( 1.71 - 4.9 ) | 2.86 ( 17.05 ) | 2.86 ( 1.84 ) | 1.52 ( 0.77 ) |
| Malignant neoplasm progression* | 13 | 9.87 ( 5.71 - 17.07 ) | 9.74 ( 102.11 ) | 9.74 ( 6.16 ) | 3.28 ( 2.51 ) |
| Fall* | 13 | 2.54 ( 1.47 - 4.39 ) | 2.52 ( 11.96 ) | 2.52 ( 1.59 ) | 1.33 ( 0.55 ) |
| Off label use | 13 | 0.91 ( 0.52 - 1.57 ) | 0.91 ( 0.12 ) | 0.91 ( 0.57 ) | -0.14 ( -0.92 ) |
| Blood creatine phosphokinase increased* | 13 | 69.88 ( 40.38 - 120.92 ) | 68.88 ( 867.25 ) | 68.68 ( 43.41 ) | 6.1 ( 5.32 ) |
| Urinary tract infection* | 12 | 3.77 ( 2.13 - 6.66 ) | 3.73 ( 24.06 ) | 3.73 ( 2.32 ) | 1.9 ( 1.09 ) |
| Product use issue* | 12 | 3.33 ( 1.88 - 5.89 ) | 3.3 ( 19.31 ) | 3.3 ( 2.05 ) | 1.72 ( 0.92 ) |
| Ageusia* | 11 | 34.75 ( 19.17 - 63.01 ) | 34.34 ( 355.66 ) | 34.29 ( 20.84 ) | 5.1 ( 4.26 ) |

Abbreviation: Asterisks (*) indicate statistically significant signals in algorithm; ROR, reporting odds ratio; PRR, proportional reporting ratio; EBGM, empirical Bayesian geometric mean; EBGM05, the lower limit of the 95% CI of EBGM; IC, information component; IC025, the lower limit of the 95% CI of the IC; CI, confidence interval; PT,preferred term; AEs, adverse events.

**Supplementary Table 5:**

**Top 20 Most Frequent Adverse Events for Sonidegib at the Preferred Term (PT) Level in Patients Aged 18 to 64 from FAERS Data**

| PT | Numbers | ROR(95%Cl) | PRR | EBGM(EBGM05) | IC(IC025) |
| --- | --- | --- | --- | --- | --- |
| Muscle spasms* | 26 | 19.87 ( 13.37 - 29.53 ) | 18.78 ( 438.63 ) | 18.76 ( 13.47 ) | 4.23 ( 3.66 ) |
| Alopecia* | 18 | 10.03 ( 6.26 - 16.08 ) | 9.67 ( 140.45 ) | 9.67 ( 6.51 ) | 3.27 ( 2.6 ) |
| Fatigue* | 17 | 2.78 ( 1.71 - 4.51 ) | 2.71 ( 18.57 ) | 2.71 ( 1.8 ) | 1.44 ( 0.74 ) |
| Myalgia* | 11 | 8.78 ( 4.83 - 15.98 ) | 8.59 ( 73.97 ) | 8.59 ( 5.21 ) | 3.1 ( 2.26 ) |
| Nausea | 9 | 1.41 ( 0.73 - 2.72 ) | 1.4 ( 1.04 ) | 1.4 ( 0.8 ) | 0.48 ( -0.44 ) |
| Basal cell carcinoma* | 9 | 87.96 ( 45.42 - 170.34 ) | 86.21 ( 756.09 ) | 85.98 ( 49.46 ) | 6.43 ( 5.5 ) |
| Vomiting* | 8 | 2.25 ( 1.12 - 4.53 ) | 2.23 ( 5.47 ) | 2.23 ( 1.24 ) | 1.16 ( 0.19 ) |
| Asthenia* | 8 | 3.29 ( 1.63 - 6.62 ) | 3.25 ( 12.52 ) | 3.25 ( 1.81 ) | 1.7 ( 0.73 ) |
| Diarrhoea | 8 | 1.73 ( 0.86 - 3.49 ) | 1.72 ( 2.45 ) | 1.72 ( 0.96 ) | 0.78 ( -0.19 ) |
| Dysgeusia* | 8 | 15.77 ( 7.84 - 31.75 ) | 15.51 ( 108.67 ) | 15.5 ( 8.63 ) | 3.95 ( 2.98 ) |
| Blood creatine phosphokinase increased* | 6 | 32.23 ( 14.4 - 72.16 ) | 31.81 ( 178.96 ) | 31.78 ( 16.19 ) | 4.99 ( 3.89 ) |
| Disease progression* | 6 | 7.52 ( 3.36 - 16.83 ) | 7.43 ( 33.46 ) | 7.43 ( 3.79 ) | 2.89 ( 1.79 ) |
| Drug ineffective | 6 | 0.71 ( 0.32 - 1.58 ) | 0.71 ( 0.72 ) | 0.71 ( 0.36 ) | -0.49 ( -1.59 ) |
| Malignant neoplasm progression* | 6 | 8.76 ( 3.91 - 19.61 ) | 8.66 ( 40.68 ) | 8.65 ( 4.41 ) | 3.11 ( 2.01 ) |
| Therapy cessation* | 6 | 11.12 ( 4.97 - 24.89 ) | 10.98 ( 54.49 ) | 10.98 ( 5.59 ) | 3.46 ( 2.36 ) |
| Ageusia* | 6 | 43.36 ( 19.36 - 97.09 ) | 42.79 ( 244.62 ) | 42.73 ( 21.77 ) | 5.42 ( 4.32 ) |
| Dyspepsia* | 5 | 7.06 ( 2.92 - 17.05 ) | 6.99 ( 25.72 ) | 6.99 ( 3.34 ) | 2.81 ( 1.62 ) |
| Pneumonia* | 5 | 2.48 ( 1.03 - 5.98 ) | 2.46 ( 4.36 ) | 2.46 ( 1.18 ) | 1.3 ( 0.11 ) |
| Death | 4 | 1.23 ( 0.46 - 3.29 ) | 1.23 ( 0.17 ) | 1.23 ( 0.54 ) | 0.3 ( -1 ) |
| Febrile neutropenia* | 4 | 8.62 ( 3.22 - 23.08 ) | 8.55 ( 26.7 ) | 8.55 ( 3.75 ) | 3.1 ( 1.8 ) |

Abbreviation: Asterisks (*) indicate statistically significant signals in algorithm; ROR, reporting odds ratio; PRR, proportional reporting ratio; EBGM, empirical Bayesian geometric mean; EBGM05, the lower limit of the 95% CI of EBGM; IC, information component; IC025, the lower limit of the 95% CI of the IC; CI, confidence interval; PT,preferred term; AEs, adverse events.

**Supplementary Table 6:**

**Top 20 Most Frequent Adverse Events for Sonidegib at the Preferred Term (PT) Level in Patients Aged Over 65 from FAERS Data**

| PT | Numbers | ROR(95%Cl) | PRR | EBGM(EBGM05) | IC(IC025) |
| --- | --- | --- | --- | --- | --- |
| Muscle spasms* | 44 | 15.24 ( 11.26 - 20.63 ) | 14.6 ( 558.02 ) | 14.57 ( 11.31 ) | 3.87 ( 3.42 ) |
| Death* | 31 | 1.49 ( 1.04 - 2.14 ) | 1.48 ( 4.9 ) | 1.48 ( 1.1 ) | 0.56 ( 0.05 ) |
| Blood creatine phosphokinase increased* | 26 | 67.65 ( 45.75 - 100.02 ) | 65.85 ( 1648.95 ) | 65.37 ( 47.13 ) | 6.03 ( 5.47 ) |
| Fatigue* | 25 | 1.88 ( 1.26 - 2.8 ) | 1.86 ( 10.05 ) | 1.86 ( 1.33 ) | 0.89 ( 0.32 ) |
| Decreased appetite* | 23 | 4.16 ( 2.75 - 6.29 ) | 4.09 ( 53.88 ) | 4.08 ( 2.89 ) | 2.03 ( 1.43 ) |
| Pneumonia* | 16 | 2.08 ( 1.27 - 3.41 ) | 2.06 ( 8.8 ) | 2.06 ( 1.36 ) | 1.04 ( 0.34 ) |
| Dysgeusia* | 16 | 12.64 ( 7.71 - 20.73 ) | 12.45 ( 168.47 ) | 12.43 ( 8.22 ) | 3.64 ( 2.93 ) |
| Nausea | 15 | 1.32 ( 0.79 - 2.19 ) | 1.31 ( 1.13 ) | 1.31 ( 0.86 ) | 0.39 ( -0.34 ) |
| Myalgia* | 15 | 5.42 ( 3.25 - 9.03 ) | 5.35 ( 53.18 ) | 5.35 ( 3.49 ) | 2.42 ( 1.69 ) |
| Asthenia | 14 | 1.67 ( 0.98 - 2.83 ) | 1.66 ( 3.69 ) | 1.66 ( 1.07 ) | 0.73 ( -0.02 ) |
| Vomiting* | 14 | 2.12 ( 1.25 - 3.6 ) | 2.11 ( 8.2 ) | 2.11 ( 1.35 ) | 1.08 ( 0.32 ) |
| Urinary tract infection* | 14 | 3.45 ( 2.03 - 5.85 ) | 3.41 ( 23.98 ) | 3.41 ( 2.19 ) | 1.77 ( 1.02 ) |
| Weight decreased* | 14 | 2.69 ( 1.58 - 4.55 ) | 2.66 ( 14.6 ) | 2.66 ( 1.71 ) | 1.41 ( 0.66 ) |
| Alopecia* | 14 | 5.61 ( 3.31 - 9.51 ) | 5.54 ( 52.23 ) | 5.54 ( 3.56 ) | 2.47 ( 1.72 ) |
| Fall | 13 | 1.33 ( 0.77 - 2.3 ) | 1.33 ( 1.06 ) | 1.33 ( 0.84 ) | 0.41 ( -0.37 ) |
| Therapy cessation* | 13 | 13.02 ( 7.53 - 22.51 ) | 12.86 ( 142.07 ) | 12.84 ( 8.12 ) | 3.68 ( 2.91 ) |
| Diarrhoea | 12 | 0.88 ( 0.5 - 1.55 ) | 0.88 ( 0.19 ) | 0.88 ( 0.55 ) | -0.18 ( -0.99 ) |
| Therapy interrupted* | 12 | 7.7 ( 4.36 - 13.62 ) | 7.62 ( 69.08 ) | 7.62 ( 4.73 ) | 2.93 ( 2.12 ) |
| Febrile neutropenia* | 11 | 8.25 ( 4.55 - 14.96 ) | 8.17 ( 69.25 ) | 8.16 ( 4.96 ) | 3.03 ( 2.19 ) |
| Sepsis* | 11 | 4.69 ( 2.59 - 8.49 ) | 4.64 ( 31.52 ) | 4.64 ( 2.82 ) | 2.21 ( 1.38 ) |

Abbreviation: Asterisks (*) indicate statistically significant signals in algorithm; ROR, reporting odds ratio; PRR, proportional reporting ratio; EBGM, empirical Bayesian geometric mean; EBGM05, the lower limit of the 95% CI of EBGM; IC, information component; IC025, the lower limit of the 95% CI of the IC; CI, confidence interval; PT,preferred term; AEs, adverse events.

**Supplementary Table 7:**

**Top 40 Positive Adverse Events Associated with Sonidegib After Excluding Concomitant Medications**

| PT | Numbers | ROR(95%CI) | PRR(χ^2^) | EBGM(EBGM05) | IC(IC025) |
| --- | --- | --- | --- | --- | --- |
| Muscle spasms | 128 | 18.53 ( 15.51 - 22.15 ) | 17.62 ( 2010.73 ) | 17.6 ( 15.17 ) | 4.14 ( 3.88 ) |
| Off label use | 83 | 1.94 ( 1.56 - 2.41 ) | 1.91 ( 36.37 ) | 1.91 ( 1.59 ) | 0.93 ( 0.61 ) |
| Alopecia | 73 | 8.12 ( 6.43 - 10.24 ) | 7.91 ( 441.74 ) | 7.9 ( 6.5 ) | 2.98 ( 2.64 ) |
| Fatigue | 72 | 2.2 ( 1.74 - 2.79 ) | 2.17 ( 45.96 ) | 2.17 ( 1.78 ) | 1.12 ( 0.77 ) |
| Death | 63 | 1.79 ( 1.39 - 2.29 ) | 1.77 ( 21.21 ) | 1.77 ( 1.43 ) | 0.82 ( 0.45 ) |
| Therapy cessation | 49 | 18.36 ( 13.84 - 24.37 ) | 18.02 ( 787.6 ) | 18 ( 14.2 ) | 4.17 ( 3.76 ) |
| Nausea | 47 | 1.54 ( 1.16 - 2.06 ) | 1.53 ( 8.82 ) | 1.53 ( 1.2 ) | 0.62 ( 0.2 ) |
| Asthenia | 43 | 2.97 ( 2.2 - 4.02 ) | 2.94 ( 55.33 ) | 2.94 ( 2.28 ) | 1.56 ( 1.12 ) |
| Ageusia | 42 | 45.84 ( 33.77 - 62.22 ) | 45.07 ( 1805.52 ) | 44.95 ( 34.81 ) | 5.49 ( 5.05 ) |
| Product dose omission issue | 41 | 3.12 ( 2.29 - 4.25 ) | 3.09 ( 58.22 ) | 3.09 ( 2.39 ) | 1.63 ( 1.18 ) |
| Myalgia | 40 | 6.37 ( 4.66 - 8.71 ) | 6.28 ( 178.13 ) | 6.28 ( 4.84 ) | 2.65 ( 2.2 ) |
| Decreased appetite | 38 | 3.98 ( 2.89 - 5.48 ) | 3.93 ( 83.33 ) | 3.93 ( 3.01 ) | 1.97 ( 1.51 ) |
| Blood creatine phosphokinase increased | 38 | 46.5 ( 33.74 - 64.1 ) | 45.8 ( 1660.88 ) | 45.67 ( 34.91 ) | 5.51 ( 5.05 ) |
| Weight decreased | 37 | 3.33 ( 2.41 - 4.61 ) | 3.3 ( 59.45 ) | 3.3 ( 2.51 ) | 1.72 ( 1.25 ) |
| Dysgeusia | 33 | 12.11 ( 8.59 - 17.08 ) | 11.96 ( 331.71 ) | 11.96 ( 8.97 ) | 3.58 ( 3.08 ) |
| Vomiting | 32 | 1.84 ( 1.3 - 2.61 ) | 1.83 ( 12.12 ) | 1.83 ( 1.37 ) | 0.87 ( 0.37 ) |
| Therapy interrupted | 29 | 9 ( 6.24 - 12.98 ) | 8.91 ( 203.75 ) | 8.9 ( 6.55 ) | 3.15 ( 2.62 ) |
| Product use issue | 29 | 2.94 ( 2.04 - 4.23 ) | 2.91 ( 36.57 ) | 2.91 ( 2.14 ) | 1.54 ( 1.01 ) |
| Arthralgia | 26 | 1.51 ( 1.03 - 2.22 ) | 1.5 ( 4.42 ) | 1.5 ( 1.09 ) | 0.59 ( 0.03 ) |
| Disease progression | 26 | 5.51 ( 3.74 - 8.11 ) | 5.46 ( 94.92 ) | 5.46 ( 3.95 ) | 2.45 ( 1.89 ) |
| Fall | 24 | 1.85 ( 1.24 - 2.76 ) | 1.84 ( 9.27 ) | 1.84 ( 1.32 ) | 0.88 ( 0.3 ) |
| Muscular weakness | 23 | 5.45 ( 3.62 - 8.22 ) | 5.41 ( 82.85 ) | 5.41 ( 3.84 ) | 2.44 ( 1.84 ) |
| Pneumonia | 22 | 1.7 ( 1.12 - 2.59 ) | 1.69 ( 6.29 ) | 1.69 ( 1.19 ) | 0.76 ( 0.16 ) |
| Malignant neoplasm progression | 21 | 4.62 ( 3 - 7.09 ) | 4.59 ( 58.97 ) | 4.58 ( 3.2 ) | 2.2 ( 1.58 ) |
| Taste disorder | 20 | 19.99 ( 12.87 - 31.04 ) | 19.83 ( 357.33 ) | 19.81 ( 13.7 ) | 4.31 ( 3.68 ) |
| Urinary tract infection | 19 | 2.75 ( 1.75 - 4.31 ) | 2.73 ( 20.92 ) | 2.73 ( 1.87 ) | 1.45 ( 0.8 ) |
| Febrile neutropenia | 17 | 6.34 ( 3.93 - 10.21 ) | 6.3 ( 75.88 ) | 6.3 ( 4.23 ) | 2.66 ( 1.97 ) |
| Basal cell carcinoma | 17 | 26.17 ( 16.24 - 42.19 ) | 26 ( 408.07 ) | 25.96 ( 17.41 ) | 4.7 ( 4.01 ) |
| Inappropriate schedule of product administration | 16 | 1.78 ( 1.09 - 2.92 ) | 1.78 ( 5.47 ) | 1.78 ( 1.18 ) | 0.83 ( 0.13 ) |
| Therapeutic product effect incomplete | 15 | 3.85 ( 2.32 - 6.4 ) | 3.83 ( 31.46 ) | 3.83 ( 2.51 ) | 1.94 ( 1.21 ) |
| Dehydration | 14 | 3.01 ( 1.78 - 5.09 ) | 3 ( 18.64 ) | 3 ( 1.93 ) | 1.58 ( 0.84 ) |
| Sepsis | 13 | 3.07 ( 1.78 - 5.29 ) | 3.06 ( 18.02 ) | 3.06 ( 1.94 ) | 1.61 ( 0.84 ) |
| Dyspepsia | 11 | 3.04 ( 1.68 - 5.5 ) | 3.03 ( 15 ) | 3.03 ( 1.85 ) | 1.6 ( 0.76 ) |
| Haemorrhage | 11 | 2.72 ( 1.5 - 4.92 ) | 2.71 ( 11.91 ) | 2.71 ( 1.65 ) | 1.44 ( 0.6 ) |
| Renal failure | 10 | 1.94 ( 1.04 - 3.6 ) | 1.93 ( 4.51 ) | 1.93 ( 1.15 ) | 0.95 ( 0.08 ) |
| Hyperkalaemia | 9 | 7.14 ( 3.71 - 13.74 ) | 7.11 ( 47.3 ) | 7.11 ( 4.11 ) | 2.83 ( 1.92 ) |
| Blood creatinine increased | 9 | 3.91 ( 2.03 - 7.52 ) | 3.9 ( 19.38 ) | 3.89 ( 2.25 ) | 1.96 ( 1.05 ) |
| Atrial fibrillation | 8 | 2.16 ( 1.08 - 4.32 ) | 2.16 ( 4.96 ) | 2.16 ( 1.21 ) | 1.11 ( 0.14 ) |
| Haematuria | 8 | 6.12 ( 3.06 - 12.25 ) | 6.1 ( 34.12 ) | 6.1 ( 3.41 ) | 2.61 ( 1.64 ) |
| Triple negative breast cancer | 8 | 371.47 ( 184.06 - 749.73 ) | 370.27 ( 2878.08 ) | 361.73 ( 201 ) | 8.5 ( 7.52 ) |

Abbreviation: ROR, reporting odds ratio; PRR, proportional reporting ratio; EBGM, empirical Bayesian geometric mean; EBGM05, the lower limit of the 95% CI of EBGM; IC, information component; IC025, the lower limit of the 95% CI of the IC; CI, confidence interval; PT,preferred term; AEs, adverse event.

**Supplementary Table 8:**

Top 20 most frequent adverse events associated with 200mg Sonidegib at the PT level

| PT | Numbers |
| --- | --- |
| Muscle spasms | 78 |
| Fatigue | 46 |
| Alopecia | 37 |
| Blood creatine phosphokinase increased | 31 |
| Decreased appetite | 24 |
| Asthenia | 22 |
| Myalgia | 22 |
| Dysgeusia | 21 |
| Diarrhoea | 20 |
| Nausea | 20 |
| Death | 16 |
| Weight decreased | 16 |
| Muscular weakness | 16 |
| Therapy cessation | 16 |
| Pneumonia | 15 |
| Basal cell carcinoma | 15 |
| Ageusia | 15 |
| Product dose omission issue | 13 |
| Arthralgia | 12 |
| Febrile neutropenia | 12 |

**Supplementary Table 9:**

Top 20 most frequent adverse events associated with 400mg Sonidegib at the PT level

| PT | Numbers |
| --- | --- |
| Vomiting | 4 |
| Disease progression | 4 |
| Malignant neoplasm progression | 4 |
| Off label use | 4 |
| Triple negative breast cancer | 4 |
| Sepsis | 3 |
| Blood creatine phosphokinase increased | 3 |
| Drug ineffective | 3 |
| Abdominal pain | 2 |
| Febrile bone marrow aplasia | 2 |
| Febrile neutropenia | 2 |
| C-reactive protein decreased | 2 |
| Gastrointestinal disorder | 2 |
| Tumour pain | 2 |
| Urinary retention | 2 |
| Cardiac failure congestive | 1 |
| Small intestinal obstruction | 1 |
| Device related infection | 1 |
| Syncope | 1 |
| Bradycardia | 1 |

**Supplementary Table 10:**

Top 20 Most Frequently Reported Adverse Events Associated with Sonidegib from 2015Q3 to 2019Q4

| PT | Numbers |
| --- | --- |
| Malignant neoplasm progression | 19 |
| Febrile neutropenia | 17 |
| Death | 12 |
| Pneumonia | 11 |
| Muscle spasms | 11 |
| Sepsis | 9 |
| Decreased appetite | 8 |
| Vomiting | 8 |
| Off label use | 8 |
| Triple negative breast cancer | 8 |
| Nausea | 7 |
| Alopecia | 7 |
| Fatigue | 7 |
| Drug ineffective | 7 |
| Asthenia | 6 |
| Atrioventricular block complete | 5 |
| Syncope | 5 |
| Arthralgia | 5 |
| Hypotension | 5 |
| Weight decreased | 5 |

Abbreviation: Q,Quarter.

**Supplementary Table 11:**

Top 20 Most Frequently Reported Adverse Events Associated with Sonidegib from 2020Q1 to 2024Q4

| PT | Numbers |
| --- | --- |
| Muscle spasms | 120 |
| Off label use | 75 |
| Alopecia | 66 |
| Fatigue | 65 |
| Death | 51 |
| Therapy cessation | 45 |
| Ageusia | 41 |
| Product dose omission issue | 41 |
| Nausea | 40 |
| Myalgia | 37 |
| Asthenia | 37 |
| Blood creatine phosphokinase increased | 36 |
| Weight decreased | 33 |
| Diarrhoea | 32 |
| Dysgeusia | 31 |
| Decreased appetite | 31 |
| Therapy interrupted | 29 |
| Product use issue | 29 |
| Drug ineffective | 26 |
| Vomiting | 24 |

Abbreviation: Q,Quarter.

**Supplementary Figure1**

Distribution of Sonidegib-Associated Adverse Events at the SOC Level from 2015Q3 to 2019Q4

**
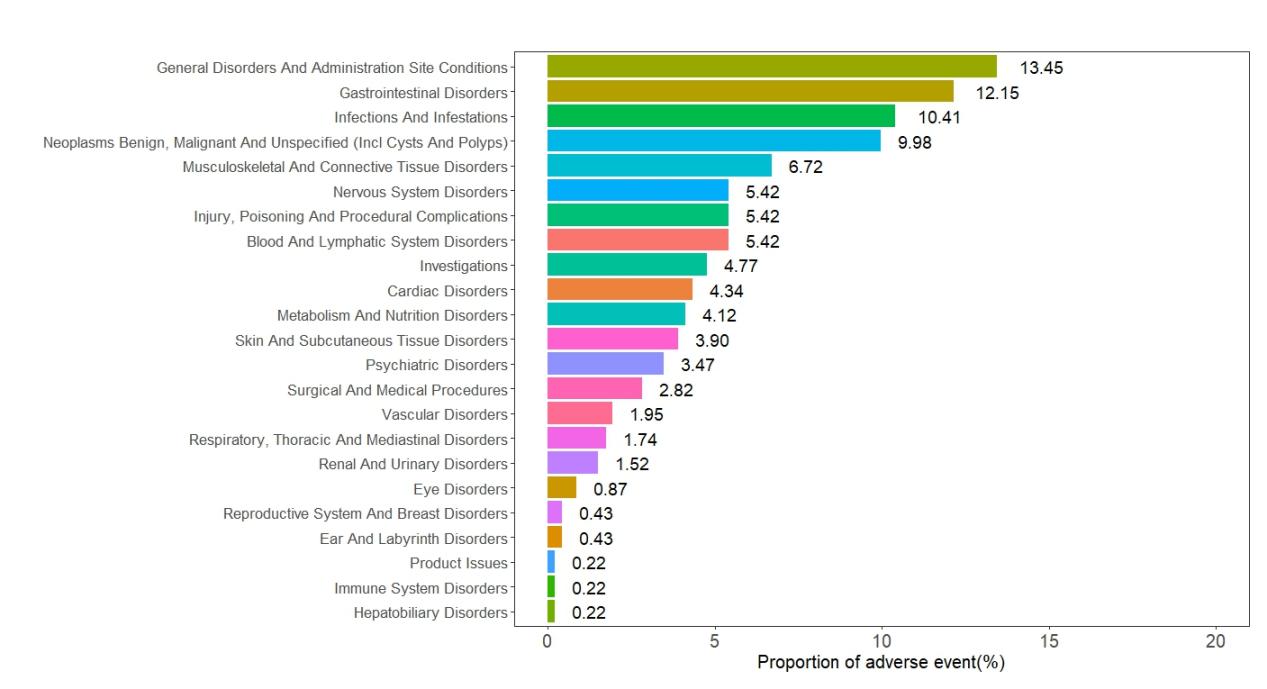
**

**Supplementary Figure2**

Distribution of Sonidegib-Associated Adverse Events at the SOC Level from 2020Q4 to 2024Q4

**
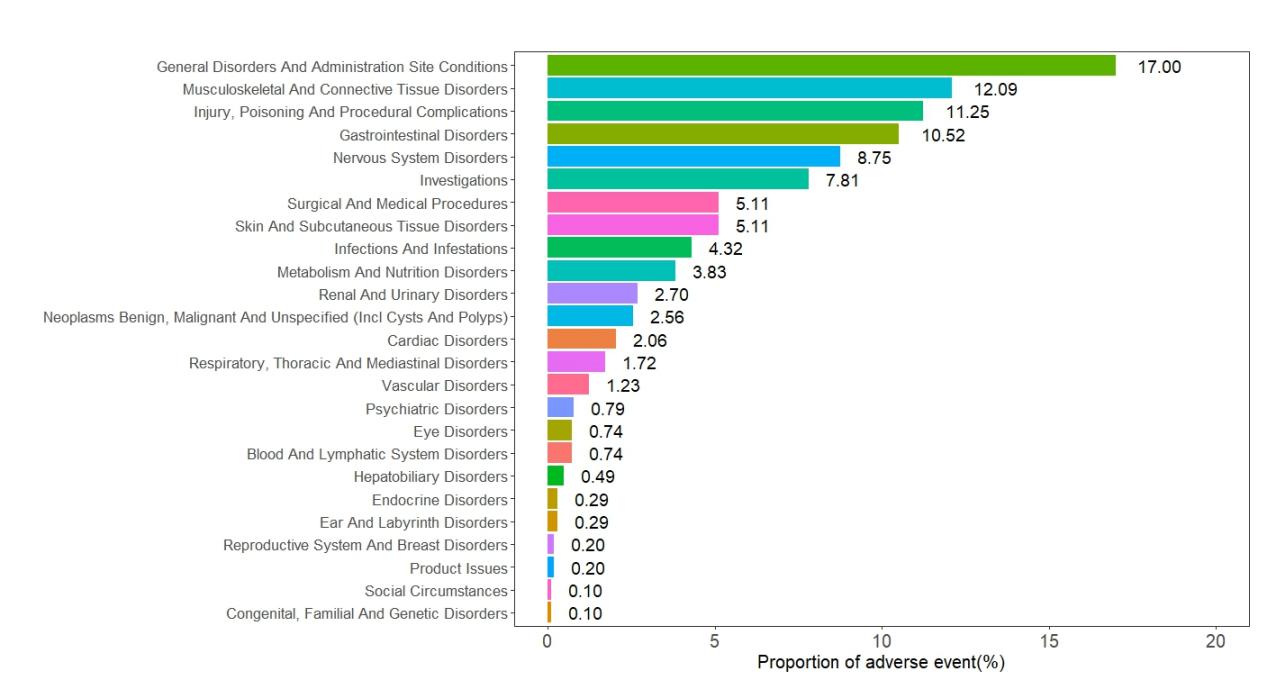
**
